# Sex Differences in Atrial Fibrillation: Evidence from Circulating Metabolites

**DOI:** 10.3390/metabo15030170

**Published:** 2025-03-02

**Authors:** Ningjing Qian, Junyan Jin, Ying Gao, Jiayi Liu, Yaping Wang

**Affiliations:** 1 Department of Cardiology, The Second Affiliated Hospital, School of Medicine, Zhejiang University, Hangzhou 310009, China; njqian@zju.edu.cn (N.Q.); junyanjin@zju.edu.cn (J.J.); 12218232@zju.edu.cn (Y.G.); 22318378@zju.edu.cn (J.L.); 2 State Key Laboratory of Transvascular Implantation Devices, Hangzhou 310009, China; 3 Heart Regeneration and Repair Key Laboratory of Zhejiang Province, Hangzhou 310009, China

**Keywords:** atrial fibrillation, sex differences, circulating metabolites, succinic acid

## Abstract

**Background**: Significant sex differences exist in atrial fibrillation (AF). Better understanding of its underlying mechanism would help AF management. This study aimed to investigate the contribution of circulating metabolites to sex differences in AF and the association between them. **Methods**: A total of 108 patients with AF were enrolled. Untargeted metabolomics were performed in plasma samples of male and female patients. Correlation analysis with clinical characteristics and Mendelian randomization were used to identify sex-specific metabolites associated with AF, which was further validated in additional patients. Transcriptomics data of the left atrium were used to investigate the molecular alteration of the left atrium responding to identified sex-specific circulating metabolites. The effect of selected sex-specific metabolites on cardiomyocytes was further investigated. **Results**: A total of 60 annotated metabolites were found with different levels between male and female patients. Among these sex-specific metabolites, three metabolites, 7-Methylguanosine, succinic acid, and N-Undecylbenzenesulfonic acid, were positively related to the left atrial remodeling. Additionally, succinic acid was significantly associated with increased risk of AF (OR = 1.26; 95% CI: 1.13 to 1.40; *p* < 0.001). And, SUCLA2, the gene of succinic acid metabolism, was significantly increased in the left atrium of male patients (fold change = 1.53; *p* = 0.008). Treatment with succinic acid led to cardiomyocyte hypertrophy and mitochondrial dysfunction. **Conclusions**: This study highlights sex differences in circulating metabolites in patients with AF and identifies the associations between sex-specific metabolites and AF. succinic acid, which is much higher in male patients, contributes to the process of AF.

## 1. Introduction

Atrial fibrillation (AF) is one of the most common arrhythmias with an increasing prevalence year by year. It affects millions of people worldwide and leads to a variety of complications, such as stroke and heart failure [1,2]. Recent epidemiological studies have highlighted significant sex differences in the prevalence, complications, and prognosis of AF [3,4]. Women are observed to have a lower risk of AF than men while they are usually more symptomatic and have more complications and poorer prognosis [5,6,7]. Despite differences in the clinical features between men and women, little is known about the correlation and contribution of sex-based differences in circulating metabolites to AF [8,9].

Circulating metabolites could provide abundant information about systemic statuses, such as metabolism and inflammation [10,11,12]. And, the incidence and process of AF could be affected by circulating metabolites in a variety of ways [13,14,15,16]. The discovery of sex-specific circulating metabolites’ association with AF might provide novel potential biomarkers and therapeutic targets for the stratification and management of AF. Untargeted metabolomics analysis could help investigate the signature of circulating metabolites in an unbiased and efficient way to clarify the potential pathogenesis and mechanism of sex differences in AF. In addition, the remodeling of the left atrium is of great importance in the process of AF [17,18,19]. The impact of circulation metabolites on left atrial remodeling is often not clear. Screening for the correlation between sex-specific circulating metabolites and left atrium remodeling could provide additional evidence for sex differences in AF.

In this study, we investigated sex differences in circulating metabolites and their associations with AF and left atrial remodeling in patients with AF. And, the association between sex-specific metabolites and left atrial remodeling was further validated in a separate population. In addition, the underlying mechanism was further explored by investigating its effect on cardiomyocytes.

## 2. Materials and Methods

### 2.1. Study Population and Design

This study was approved by the Ethics Committee of the Second Affiliated Hospital of Zhejiang University School of Medicine. Patients with ages between 18 and 85 years who underwent radio-frequency ablation for AF for the first time in the Second Affiliated Hospital of Zhejiang University, School of Medicine, were recruited. Eligible patients were further filtered by the following exclusion criteria: valvular diseases, acute coronary syndrome, malignant tumor, and severe liver or renal dysfunction. For the discovery group, a total of 34 patients, including 18 male patients and 16 female patients, were enrolled between June 2023 and September 2023 to collect blood samples for untargeted metabolomics analysis. After that, an additional 74 matched patients, including 35 male patients and 39 female patients, were enrolled between October 2023 and April 2024 to collect blood samples and measure the selected metabolite level for validation. All participants provided written informed consent. All blood samples were collected in a fasting state.

### 2.2. Data Collection

The clinical characteristics were collected at baseline from the electronic medical record (EMR) system. All participants underwent echocardiography prior to radio-frequency ablation by an independent cardiologist. And, the LA-ap (anterior and posterior diameter of left atrium) data were collected to evaluate the structural remodeling of the LA.

### 2.3. Untargeted Metabolomics Analysis

Plasma samples were separated by centrifugation at 4 °C and 1000× *g* for 10 min and stored at −80 °C. The collected plasma samples were thawed and metabolites were extracted with 80% methanol buffer. The samples were redissolved with 100 μL 80% methanol and stored at −80 °C prior to the LC-MS analysis. Then, all samples were acquired by the LC-MS system according to the instrument specification. The UltiMate 3000 UPLC System (Thermo Fisher Scientific, Bremen, Germany) was used for chromatographic separations and the ACQUITY UPLC T3 column was used for the reverse-phase separation. A high-resolution tandem mass spectrometer, Q-Exactive, was used to detect metabolites eluted from the column. Q-Exactive was operated in both positive and negative ion modes. Precursor spectra (70–1050 *m*/*z*) were collected at 70,000 resolutions to hit an AGC target of 3 × 10^6^. The maximum injection time was set to 100 ms. A top-3 configuration to acquire data was set in the DDA mode. Fragment spectra were collected at 17,500 resolutions to hit an AGC target of 1 × 10^5^ with a maximum injection time of 80 s. In order to evaluate the stability of the LC-MS during the whole acquisition, a quality control sample (pool of all samples) was acquired after every 10 samples.

### 2.4. Metabolomic Data Analysis

The identified metabolites were annotated by matching the exact molecular mass data (*m*/*z*) in the KEGG and HMDB databases. Partial least squares discriminant analysis (PLS-DA) was performed in metaX (1.4.19). Student’s *t*-test was performed to detect differential metabolites between male and female patients. The MetaboAnalyst 6.0 database was further used to analyze the enrichment of differential metabolites and test potential causal relationships between the selected metabolites and AF via mGWAS by the two-sample Mendelian randomization method.

### 2.5. Succinic Acid Assay

The levels of succinic acid in plasma samples were evaluated by the succinic Acid Colorimetric Assay Kit (Elabscience Biotechnology Co., Ltd., Wuhan, China). The analytical sensitivity was specified as 0.043 mmol/L.

### 2.6. Transcriptomics Sequencing Data Acquisition and Processing

The dataset’s original files were downloaded from the NCBI GEO database and only datasets providing sex information were taken in account. GSE41177 and GSE79768 were finally included for further analysis. For left atrium samples, a total of 9 patients with SR and 23 patients with AF were included. The information of the samples is shown in Table A1. The datasets were from the microarray platform of [HG-U133_Plus_2] Affymetrix Human Genome U133 Plus 2.0 Array. And, the limma R software package (3.6.1) was used for background correction, quartile standardization, and probe summary [20,21].

### 2.7. Screening of Differential Expression Genes (DEGs)

The limma R package was used to screen DEGs [20]. |log2FC| > 1 and *p* < 0.05 were used as the thresholds to determine DEGs, except where otherwise stated.

### 2.8. Functional Enrichment and Pathway Enrichment Analysis

The Gene Ontology (GO) database was applied to analyze the pathway enrichment of DEGs via the human genome annotation package “org. HS. eg.db” for Entrez ID convention and “Clusterprofiler” KEGG and GO enrichment analysis packages for enrichment analysis in R software (4.1.0) [22,23,24,25]. The visualization of significant pathways was based on a *p*-adjusted value < 0.05 and count of DEGs.

### 2.9. Cell Culture and In Vitro Treatment

H9C2 rat cardiomyocytes were maintained in DMEM (Gibco; Thermo Fisher Scientific, Inc., USA) supplemented with 10% FBS (Life iLab, Co., Ltd., Shanghai, China) at a humidified atmosphere of 5% CO_2_ at 37 °C. The cells were treated with or without 0.3 mM sodium succinate (Aladdin, Co., Ltd., Shanghai, China) for 48 h before harvesting.

### 2.10. Western Blot

Proteins were extracted from cultured cells after rinsing with PBS via RIPA solution and quantified by the BCA Protein Assay Kit (Thermo Fisher Scientific, Inc., USA). The quantified proteins were loaded on 10% SDS/PAGE gel and transferred onto polyvinylidene difluoride membranes. The membranes were blocked with 5% skimmed milk and incubated with specific primary antibodies at 4 °C overnight. The primary antibody was ANP (Santa cruz). Then, the membranes were incubated with a secondary antibody: HRP Conjugated Rabbit anti-Mouse IgG polyclonal Antibody. Immunoblots were quantified by ImageJ Lab software (1.54).

### 2.11. Measurement of Mitochondrial Membrane Potential

The mitochondrial membrane potential of the cells was evaluated by an enhanced mitochondrial membrane potential assay kit with JC-1 (Beyotime, Co., Ltd., Shanghai, China). In brief, the cells were incubated with JC-1 working buffer after rinsing with PBS at 37 °C for 20 min and then washed with JC-1 staining buffer two times. The mitochondrial membrane potential was detected by a fluorescence microscope and quantified by the fluorescence intensity.

### 2.12. Measurement of Reactive Oxygen Species (ROS)

ROS generation was detected by DCFH-DA staining (Beyotime, Co., Ltd., Shanghai, China). In brief, the cells were incubated with DCFH-DA after rinsing with PBS at 37 °C for 30 min in the dark and then washed with PBS three times. ROS generation was detected by a fluorescence microscope and quantified by the fluorescence intensity.

### 2.13. Statistical Analysis

Clinical characteristics were quantitatively represented as the mean ± standard deviation (SD) or median values (interquartile range), and categorical variables were represented as percentages. Student’s *t*-test or the Mann–Whitney U test were performed to identify the differences between groups. Pearson’s analysis was performed to assess the correlations of metabolites and the LA-ap. Univariate and multivariate linear regression were further used to test the contribution of metabolites to the LA-ap. *p* < 0.05 was considered a statistically significant difference.

## 3. Results

### 3.1. Sex Difference in Circulating Metabolites’ Signature Between Male and Female Patients with AF

**Figure 1:**
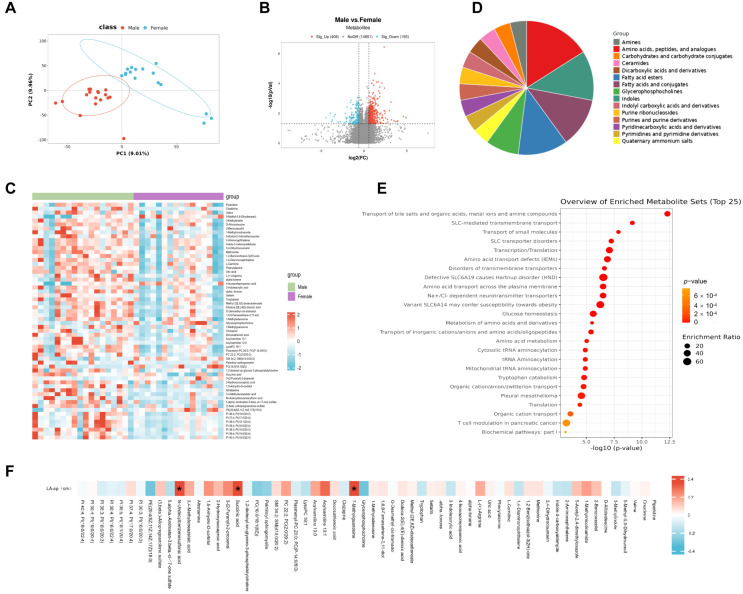
Sex difference in circulating metabolite signatures between male and female patients with atrial fibrillation. (**A**) OPLS-DA analysis of samples from male patients and female patients. (**B**) Volcano plot of all differential metabolites between male patients and female patients. (**C**) Heatmap of annotated differential metabolites between male patients and female patients. (**D**) Metabolite set enrichment of annotated differential metabolites. (**E**) Pathway enrichment of annotated differential metabolites based on RaMP-DB. (**F**) Correlation between annotated differential metabolites and anterior and posterior diameter of left atrium (LA-ap). *, *p* < 0.05.

To assess differences in circulating metabolite patterns between male and female patients with AF, untargeted metabolomics analysis was performed on blood plasma samples of 18 male patients and 16 female patients with AF to characterize and quantify the metabolites. The clinical characteristics of the 18 male patients and 16 female patients with AF are summarized in Table 1. An OPLS-DA model built numerous times (*n* = 200) revealed a significant difference between male patients with AF and female patients with AF (Figure 1A). The overall distribution of differential metabolites is visualized as volcano plots (Figure 1B). After annotating the differential metabolites from reliable databases, a total of 60 metabolites with *p* < 0.05 were identified as being significantly different between male and female patients, with 53 metabolites higher in male patients and 7 metabolites higher in female patients (Figure 1C). As shown in Figure 1D, the 60 metabolites mainly belonged to four classes, including amino acids, peptides and their analogs, indoles, fatty acids and their conjugates, and fatty acid esters. Metabolite set enrichment analysis was performed to investigate potential perturbed pathways. The differential metabolites were mainly involved in the transport of bile salts, organic acids, metal ions, and amine compounds, SLC-mediated transmembrane transport, the transport of small molecules, SLC transporter disorders, and transcription/translation (Figure 1E). To further explore the contribution of differential metabolites to sex difference in AF, correlation analysis was used to screen the relationship between metabolites and the structural remodeling index of the left atrium, LA-ap. Among the 60 differential metabolites, three metabolites, including 7-Methylguanosine, succinic acid, and N-Undecylbenzenesulfonic acid, were identified to have significant associations with the LA-ap (Figure 1F).

### 3.2. Sex-Specific Metabolites Involved in Structural Remodeling of Left Atrium

**Figure 2:**
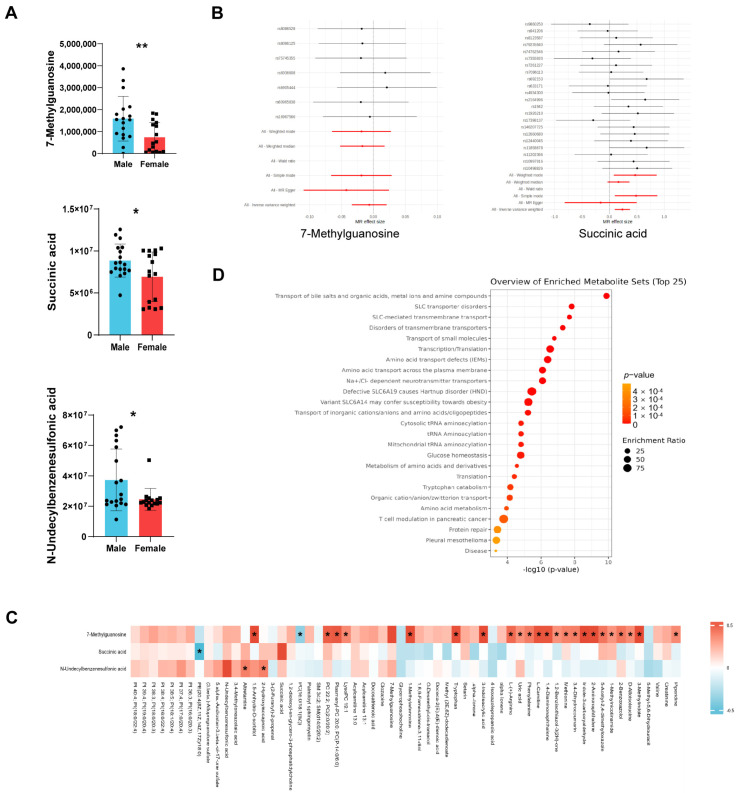
Sex-specific metabolites involved structural remodeling of left atrium during atrial fibrillation. (**A**) Levels of three sex-specific metabolites, 7-Methylguanosine, succinic acid, and N-Undecylbenzenesulfonic acid, in male and female patients with atrial fibrillation. (**B**) Forest plot of causality of metabolites 7-Methylguanosine and succinic acid on atrial fibrillation. (**C**) Heatmap of correlation between selected metabolites and other annotated differential metabolites. (**D**) Pathway enrichment of metabolites related to 7-Methylguanosine based on RaMP-DB. *, *p* < 0.05; **, *p* < 0.01.

The plasma levels of the three sex-specific metabolites involved in the structural remodeling of the left atrium were compared between male and female patients. As shown in Figure 2A, compared to female patients, male patients with AF had remarkably higher average levels of 7-Methylguanosine, succinic acid, and N-Undecylbenzenesulfonic acid. Univariate linear regression analysis revealed that in the unadjusted model, 7-Methylguanosine, succinic acid, and N-Undecylbenzenesulfonic acid were, respectively, positively correlated with the LA-ap (β = 1.64; 95% CI: 0.11 to 3.16; *p* = 0.036; β = 0.59; 95% CI: 0.03 to 1.15; *p* = 0.040; β = 0.09; 95% CI: 0.01 to 0.18; *p* = 0.040). The relationships between 7-Methylguanosine, succinic acid, N-Undecylbenzenesulfonic acid, and the LA-ap remained robustly significant in the fully adjusted model (Table 2). To further investigate the role of these sex-specific metabolites in AF, the potential causal relationships between the selected metabolites and AF were tested via mGWAS by the two-sample Mendelian randomization method (Figure 2B). Notably, succinic acid was significantly associated with increased risk of AF (OR = 1.26; 95% CI: 1.13 to 1.40; *p* < 0.001). For 7-Methylguanosine, analysis based on 7-Methylguanine found with no causal effect on AF. N-Undecylbenzenesulfonic acid was not identified in the database. Since metabolites could have interaction effects on one another and could interconvert under certain conditions, correlations between the levels of three sex-specific metabolites and the other differential metabolites were examined (Figure 2C). The level of 7-Methylguanosine had abundant correlations with several other metabolites. And, these metabolites related to 7-Methylguanosine were enriched in pathways mainly related to transmembrane transport and tRNA aminoacylation (Figure 2D). The level of succinic acid only had a negative correlation with PE(20:4(8Z,11Z,14Z,17Z)/18:0). And, the level of N-Undecylbenzenesulfonic acid had positive correlations with altretamine and 2-Hydroxyisocaproic acid.

### 3.3. Sex-Specific Metabolites Were Associated with Transcriptomic Signature of Left Atrium

**Figure 3:**
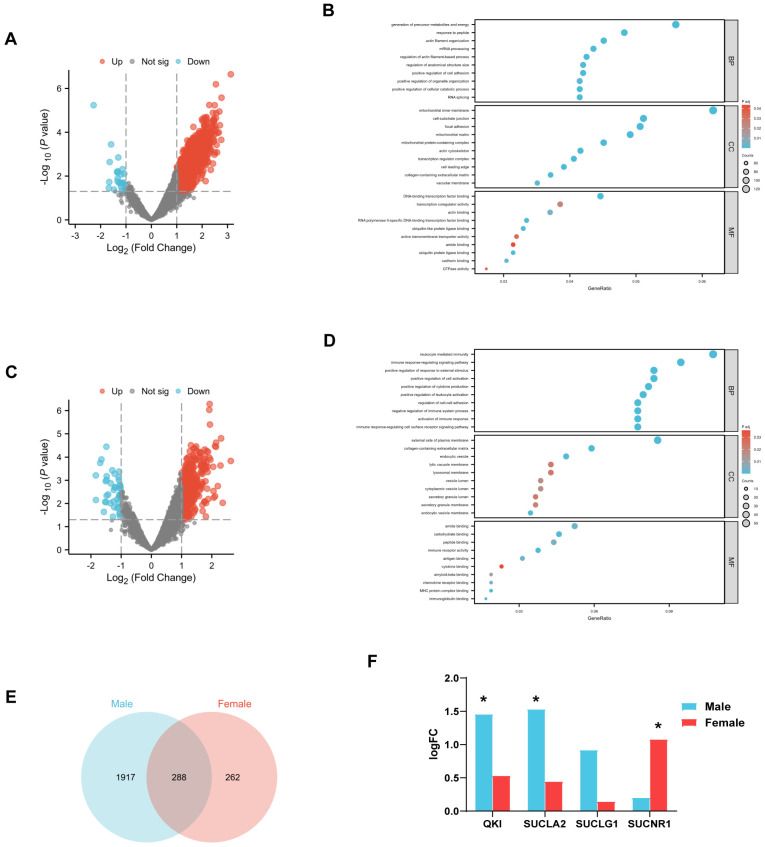
Sex differences in transcriptomic signature of left atrium and expression of genes related to sex-specific metabolites. (**A**) Volcano plot of gene expressions for left atrium in male patients with screening criteria of |log2 FC|≥ 1 and *p* value < 0.05 for different expression genes (DEGs) between atrial fibrillation (AF) group and sinus rhythm (SR) group. (**B**) Gene Ontology pathway enrichment of DEGs in male patients. (**C**) Volcano plot of gene expressions for left atrium in female patients with screening criteria of |log2 FC|≥ 1 and *p* value < 0.05 for DEGs between AF group and SR group. (**D**) Gene Ontology pathway enrichment of DEGs in female patients. (**E**) DEGs of left atrium in male patients between AF and SR group intersect with DEGs of left atrium in female patients. (**F**) Fold change in expression of genes related to selected metabolites in male and female patients between AF and SR group, separately. *, *p* < 0.05.

To further explore the influence of the sex-specific metabolites on sex differences in AF, the transcriptomic signature of the left atrium in male patients and female patients with AF compared to those with sinus rhythm (SR) were analyzed, respectively. For male patients, 2205 DEGs were observed between the AF and SR groups under the settings of |log2FC| > 1 and *p* < 0.05. Among them, 2179 were upregulated DEGs and 26 were downregulated DEGs (Figure 3A). Consistent with the high levels of series of metabolites including amino acids, peptides and their analogs, indoles, fatty acids and their conjugates, and fatty acid esters, GO analysis highlighted that the DEGs of the left atrium in male patients with AF were mainly involved in metabolic process. Mitochondrial-related pathways were represented multiple times, along with the generation of precursor metabolites and energy and the positive regulation of cellular catabolic process (Figure 3B). In comparison, there were 550 DEGs between the AF and SR groups in female patients, of which 508 DEGs were upregulated and 42 DEGs were downregulated in the AF group (Figure 3C). GO analysis showed that the DEGs of the left atrium in female patients were concentratedly involved in the immune response and regulation based on immune cells, cytokines, and chemokines (Figure 3D), which suggested the probable influence of metabolites like Glycerophosphocholine and phosphatidylcholine, which were higher in female patients with AF. And, it is worth noting that only 288 DEGs were commonly regulated in male and female patients when comparing the AF group and SR group. However, there were 1917 DEGs only specifically regulated in male patients and 262 DEGs specifically regulated in female patients (Figure 3E). Considering the significant association between 7-Methylguanosine, succinic acid, and AF, the expression of genes related to the metabolism of them were further compared (Figure 3F). QKI was significantly increased in the left atrium of male patients with AF compared those with SR, which was reported to modulate 7-Methylguanosine modifications in tRNA. The expression of QKI was not significantly altered between the AF and SR groups in female patients. The gene related to the metabolism of succinic acid, SUCLA2, was significantly increased in the left atrium of male patients with AF compared those with SR (fold change = 1.53; *p* = 0.008), while it had little change in female patients. SUCNR1 was obviously raised in the left atrium of female patients with AF but not in male patients.

### 3.4. Validation of Sex Difference in Plasma Levels of Succinic Acid and Its Involvement with Left Atrium Remodeling

**Figure 4:**
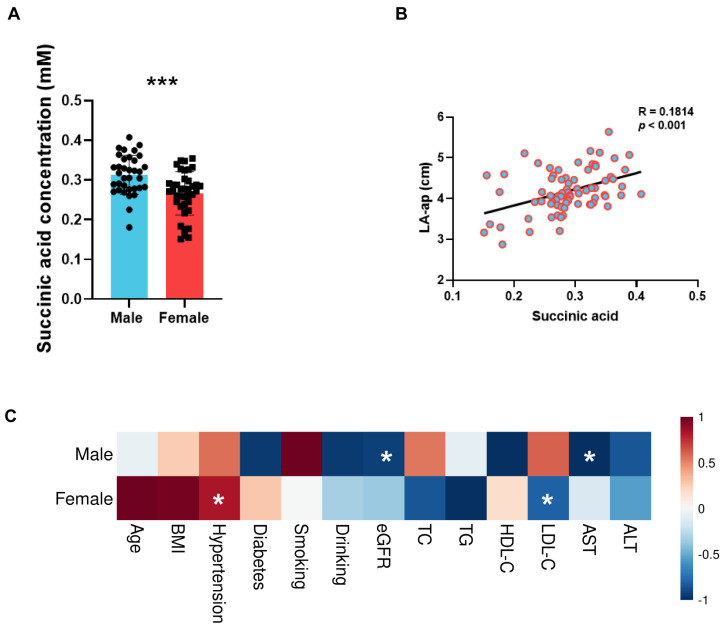
Validation of sex difference in plasma levels of succinic acid and its involvement with left atrium remodeling. (**A**) Levels of succinic acid in plasma of male and female patients with atrial fibrillation. (**B**) Scatter diagram of correlation between succinic acid levels and anterior and posterior diameter of left atrium (LA-ap). (**C**) Heatmap of correlation between succinic acid and clinical characteristics in male and female patients. BMI, body mass index; eGFR, estimated glomerular filtration rate; TC, total cholesterol; TG, triglyceride; HDL-C, high-density lipoprotein cholesterol; LDL-C, low-density lipoprotein cholesterol; AST, aspartate aminotransferase; ALT, alanine aminotransferase. *, *p* < 0.05; ***, *p* < 0.001.

Considering the results of the correlation and mGWAS analysis, the plasma level of succinic acid was evaluated in an additional 74 patients with AF. The clinical characteristics of the 74 patients, including 35 male patients and 39 female patients, are summarized in Table A2. Consistently, the plasma concentration of succinic acid was higher in male patients with AF than that in female patients (0.31 ± 0.05 mM vs. 0.27 ± 0.05 mM; *p* < 0.001) (Figure 4A). And, importantly, it was proven that the plasma concentration of succinic acid was positively correlated with the LA-ap (Figure 4B). Further investigation showed that the plasma levels of succinic acid were associated with different clinical characteristics between sexes (Figure 4C). In male patients, a lower estimated glomerular filtration rate (eGFR) (r = −0.40; *p* = 0.020) and aspartate aminotransferase (AST) levels (r = −0.40; *p* = 0.017) were related to elevated levels of succinic acid. Meanwhile, in female patients, hypertension (r = 0.33; *p* = 0.043) and lower levels of low-density lipoprotein cholesterol (LDL-C) (r = −0.32; *p* = 0.048) were associated with increased levels of succinic acid.

### 3.5. Succinic Acid-Induced Cardiomyocyte Hypertrophy and Mitochondrial Dysfunction

**Figure 5:**
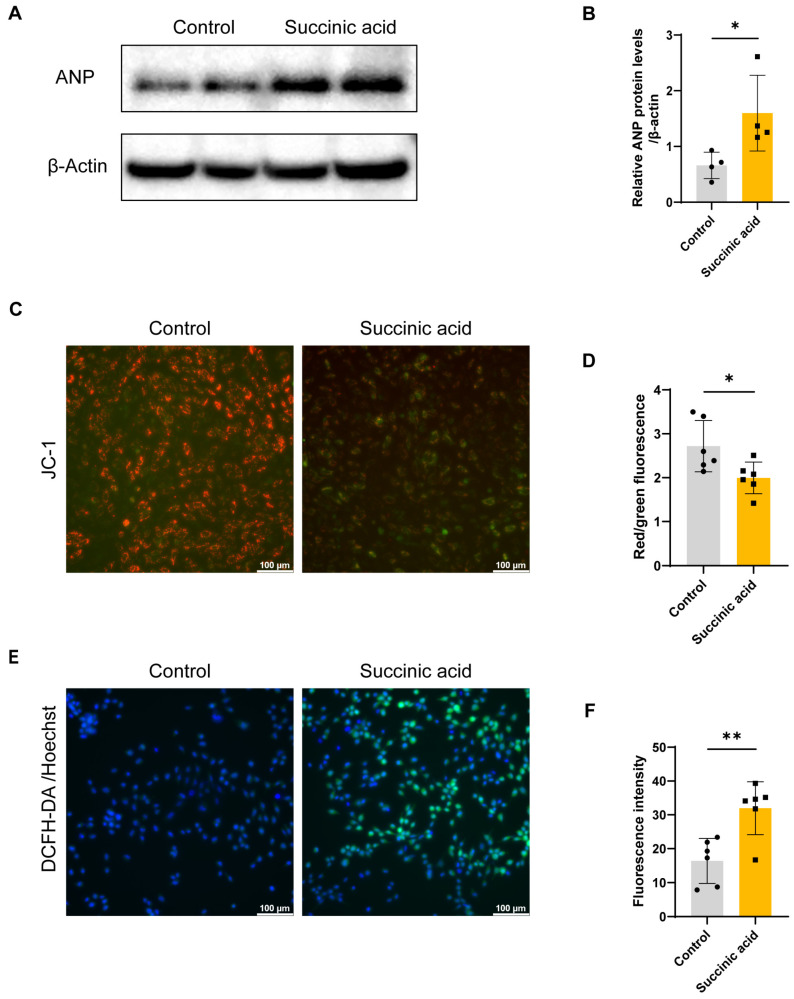
Succinic acid-induced cardiomyocyte hypertrophy and mitochondrial dysfunction. (**A**, **B**) Expression of hypertrophy marker ANP was assessed by Western blot in H9C2 cardiomyocytes treated with succinic acid and control group (*n* = 4). (**C**, **D**) JC-1 staining images and quantification results of H9C2 cardiomyocytes treated with succinic acid and control group (*n* = 6). The red fluorescence represents JC-1 aggregates and the green fluorescence represents JC-1 monomer. (**E**, **F**) ROS staining images and quantification results of H9C2 cardiomyocytes treated with succinic acid and control group (*n* = 6). *, *p* < 0.05; **, *p* < 0.01.

In view of the crucial role of succinic acid in sex differences in AF and the structural remodeling of the left atrium, the impact of succinic acid on cardiomyocytes was extensively investigated. H9C2 cardiomyocytes were treated with average circulating concentrations under pathological conditions. With the treatment of succinic acid, there was a remarkable increase in the expression of the hypertrophic marker ANP (Figure 5A,B). To determine whether succinic acid induced cardiomyocyte hypertrophy by regulating mitochondria function, H9C2 cardiomyocytes were labeled by JC-1 after treatment with succinic acid. A significant decrease in mitochondrial membrane potential based on the analysis of the red/green fluorescence ratio was detected in the group treated with succinic acid (Figure 5C,D). And, ROS staining further revealed that succinic acid significantly increased oxidative stress in cardiomyocytes (Figure 5E,F).

## 4. Discussion

This study is the first to represent a comprehensive sex-specific circulating metabolite signature analysis in patients with AF and identify the sex-specific metabolites contributing to AF. The sex-specific metabolite succinic acid is observed with a higher plasma level in male patients than female patients. It is related to the structural remodeling of the left atrium and the risk of AF. Analysis of left-atrium transcriptomics further reveals that the distinct molecular signatures of atrial remodeling between male and female patients are associated with circulating metabolite differences. Cardiomyocyte treatment with succinic acid in vivo suggests that succinic acid might be involved in the progress of AF by promoting cardiomyocyte hypertrophy and influencing mitochondrial function.

Accumulating evidence supports the effect of sex on AF since significant differences are observed in prevalence, recurrence, epidemiology, and clinical outcomes between male and female patients [26,27,28,29]. Previous studies have suggested that male patients are exposed to a relatively higher risk of AF than female patients. And, male patients with AF show a worse response to antiarrhythmic drugs. However, female patients are more likely to suffer complications of AF and have worse outcomes after catheter ablation [4,30,31,32]. Stronger fibrotic responses are usually detected in the atrium of male patients while the role of inflammation is prominent in the progress of AF in female patients [33,34]. Our work tries to provide insight from metabolomics to understand the sex differences in AF. It was observed that male patients with AF had higher plasma levels of metabolites like amino acids, peptides and their analogs, and fatty acids than females, which could work as energy substrates. Metabolism and energy supplements are supposed to be critical determinants of electrical and structural remodeling in AF. The electrical and contractile activity of cardiomyocytes consume energy or, in other words, adenosine tri-phosphate (ATP). The high beating rates during AF demand more ATP supplements [35]. When the transcriptomic signature of the left atrium in male patients was analyzed separately, it showed that several metabolic-associated genes were significantly upregulated, with large fold changes and mitochondrial-related pathways represented multiple times. This highlighted the involvement of upregulated energy supplements and the consumption of the left atrium in male patients with AF, while these alterations were not as strong in the analysis of the left atrium in female patients. These findings suggest that certain circulating metabolites’ alteration might reflect and influence metabolic adaptation in the atrium.

Previous studies have suggested that inflammation might be deeply involved in the trigger and development of AF. And, clinical studies have shown the association of AF and infectious diseases and autoimmune disease such as sepsis, myocarditis, and rheumatic heart disease [36,37,38,39]. Our metabolomics analysis showed that the levels of Glycerophosphocholine, phosphatidylcholine, and several metabolites of the same class were much higher in female patients but lower in male patients. Previous studies have reported that the decrease in phosphatidylcholine is related to the raised risk of AF [40]. Phosphatidylcholine is involved in anti-inflammatory processes and lower levels of phosphatidylcholines are associated with insufficient capacity in the response to inflammation. Consistent with this notion, we observed more obviously striking rises in inflammation-related transcripts and pathways in the left atrium of female patients with AF. These findings further highlight the association between circulating metabolites and atrial remodeling in AF from the perspective of inflammation, especially in female patients.

Notably, the sex-specific differential metabolite succinic acid was found to be positively associated with the structural remodeling index of the left atrium, the LA-ap, and the increased risk of AF. And, the effect of succinic acid on the structural remodeling of the left atrium was independent of heart failure-related left ventricular remodeling. The role of succinic acid as the intermediate metabolite of the citric acid cycle has been attached to great importance in previous studies [41]. For example, during ischemia and hypoxia, succinic acid accumulates in mitochondria and is further oxidized by succinate dehydrogenase, promoting the production of reactive oxygen species. The reactive oxygen in excess would induce cardiomyocyte injury. However, the role of exogenous or circulating succinic acid has not been well investigated in the heart. Consistently with our results, the PREDIMED study demonstrated that the elevated plasma level of succinic acid is associated with a higher risk of AF [42]. Our study further explored the potential underlying mechanism of increased plasma levels in succinic acid for the structural remodeling of the left atrium and AF. Our results showed that exogenous succinic acid treatment, serving a similar function to endogenous accumulation, leads to mitochondrial dysfunction and increased oxidative stress in cardiomyocytes. And, it is known that the increased generation of ROS and disrupted mitochondrial function exacerbates pathological cardiomyocyte hypertrophy [43], which are critical features during the disease progress of AF. In addition, research in the field of cancer has shown that succinic acid accumulation in microenvironments could enhance cancer cell migration and invasion and promote inflammatory infiltration [44,45]. As mentioned above, inflammation plays a critical role in the triggering and development of AF. In this way, increased levels of succinic acid may also contribute to inflammatory infiltration via immune cell recruitment and the secretion of inflammatory factor during AF, which need to be explored in further work. Moreover, studies on the regulation of sepsis have shown that increased succinic acid levels could probably activate intracellular calcium signal transduction and regulate protein function by succinylation [46]. Calcium signaling is thought to be one of the most important contributors to AF promotion. Abnormal calcium signaling could lead to reduced action-potential duration and promote beat-to-beat alternation in action-potential duration, which favors reentry and spontaneous ectopy due to delayed afterdepolarizations. And, these drive atrial electrical remodeling during AF [47]. Given this, succinic acid-caused cardiomyocyte and atrial adaption might be the potential mechanism of sex differences in AF.

Previous studies show that in healthy patients, the circulating levels of succinic acid are relatively very low, even undetectable [48]. Our results showed that during AF, circulating levels of succinic acid rose to millimolar levels, at an average of 0.3 mM. Although the data are preliminary, they suggest that an elevated circulating succinic acid level might serve as a potential marker for AF. The monitoring of succinic acid levels may help to establish a new risk prediction model for AF. And, monoclonal antibodies or inhibitors targeting succinic acid receptors or metabolic processes may become potential therapeutic agents for AF. Further investigating the association between plasma levels in succinic acid and clinical characteristics, we found that succinic acid showed correlation with different clinical characteristics in male and female patients. Circulating succinic acid can be derived from multiple sources, including endogenous cellular metabolism and the metabolic activity of gut microbiota, which is subsequently absorbed into the blood. The changes in specific gut microbiota involved in succinic acid production and consumption can have a large impact on the circulating levels of it, while the balance of a specific gut microbiota could be influenced by various factors, such as diet, temperature, and exercise [49]. Therefore, the factors contributing to sex differences in succinic acid levels could be diversified. Further studies deeply looking for the factors leading to the raise of succinic acid levels would help better understanding the sex differences in it and its role in AF.

There are some limitations in our study. The etiology and pathological physiology of AF are complex and the underlying mechanism might differ from nonvalvular AF and valvular AF. Our samples only included patients with non-valvular AF. The results should be further validated when extrapolating to populations of valvular AF. And, all patients in the study were Asian patients. Therefore, caution is warranted when extrapolating our study findings to the broader AF population. And, further long-term and functional studies are necessary to explore and validate the impact of succinic acid on the prognosis and complications of AF. Additional work with a large number of patients in multiple dimensions, such as spatial transcriptomics and inflammatory cytokines in peripheral blood evaluation, will be needed to confirm our work and evaluate its clinical translatability.

## 5. Conclusions

We established a sex stratification in metabolomics analysis to provide molecular insights into sex differences in AF. The sex-specific differential metabolite succinic acid, which is much higher in male patients with AF than female patients, is independently associated with the structural remodeling of the left atrium during AF and the risk of AF. The potential underlying mechanism is succinic acid-induced cardiomyocyte adaption during AF.

## Figures and Tables

**Table 1:** Clinical characteristics of patients for metabolomic analysis.

Characteristic	Sex		*p*
	Male, *n* = 18	Female, *n* = 16	
Age	65.44 ± 8.59	65.63 ± 9.92	ns
BMI	24.38 ± 3.60	24.52 ± 3.24	ns
Hypertension			ns
No	12 (66.67%)	6 (37.50%)	
Yes	6 (33.33%)	10 (62.50%)	
Diabetes			ns
No	16 (88.89%)	12 (75.00%)	
Yes	2 (11.11%)	4 (25.00%)	
Smoking			<0.001
No	5 (27.78%)	16 (100.00%)	
Yes	13 (72.22%)	0 (0.00%)	
Drinking			0.008
No	9 (50.00%)	15 (93.75%)	
Yes	9 (50.00%)	1 (6.25%)	
LVEF (%)	63.06 ± 7.22	64.53 ± 6.90	ns
IVSd (cm)	0.98 ± 0.13	0.94 ± 0.17	ns
LVIDd (cm)	4.73 ± 0.44	4.44 ± 0.35	0.041
LVPWd (cm)	0.92 ± 0.12	0.91 ± 0.12	ns
LVIDs (cm)	3.16 ± 0.40	2.88 ± 0.34	0.036
AO-STJ (cm)	2.93 ± 0.36	2.71 ± 0.24	0.049
LA-ap (cm)	4.10 ± 0.39	4.01 ± 0.49	ns
eGFR, mL/(min*1.73 m^2^)	80.96 ± 14.05	80.40 ± 29.07	ns
TC, mmol/L	4.17 ± 0.67	4.62 ± 1.10	ns
TG, mmol/L	1.43 ± 0.74	1.52 ± 0.66	ns
HDL-C, mmol/L	1.26 ± 0.26	1.27 ± 0.28	ns
LDL-C, mmol/L	2.17 ± 0.50	2.53 ± 0.91	ns
AST, U/L	19.50 (16.50, 27.00)	15.00 (10.25, 26.50)	ns
ALT, U/L	24.50 (18.75, 31.00)	22.00 (19.25, 26.50)	ns

ns, not significant; BMI, body mass index; LVEF, left ventricular ejection fraction; IVSd, end-diastolic interventricular septum thickness; LVIDd, left ventricular end-diastolic inner diameter; LVPWd, left ventricular end-diastolic posterior wall thickness; LVIDs, left ventricular end-systolic inner diameter; AO-STJ, aorta sinotubular junction; LA-ap, anterior and posterior diameter of left atrium; eGFR, estimated glomerular filtration rate; TC, total cholesterol; TG, triglyceride; HDL-C, high-density lipoprotein cholesterol; LDL-C, low-density lipoprotein cholesterol; AST, aspartate aminotransferase; ALT, alanine aminotransferase.

**Table 2:** Linear regression analysis of sex-specific metabolites and anterior and posterior diameter of left atrium.

LA-ap	Model 1 ^A^		Model 2 ^B^		Model 3 ^C^	
	β (95% CI)	*p*	β (95% CI)	*p*	β (95% CI)	*p*
7-Methylguanosine	1.64 (0.11, 3.16)	0.036	1.67 (0.03, 3.31)	0.047	2.35 (0.52, 4.18)	0.014
Succinic acid	0.59 (0.03, 1.15)	0.040	0.63 (0.04, 1.21)	0.036	0.76 (0.05, 1.46)	0.036
N-Undecylbenzenesulfonic acid	0.09 (0.01, 0.18)	0.040	0.11 (0.01, 0.19)	0.030	0.12 (0.01, 0.24)	0.041

^A^, unadjusted model; ^B^, adjusted for age and left ventricular ejection fraction; ^C^, adjusted for age, left ventricular ejection fraction, sex, BMI, smoking, drinking, and eGFR. LA-ap, anterior and posterior diameter of left atrium; BMI, body mass index; eGFR, estimated glomerular filtration rate.

## Data Availability

Data will be made available upon reasonable request. The data are not publicly available due to ethical reasons.

## Abbreviations

The following abbreviations are used in this manuscript:

AF	atrial fibrillation
SR	sinus rhythm
LA	left atrium
DEGs	differential expression genes

## Appendix A

### Appendix A.1

**Table A1:** Clinical characteristics of patients with sinus rhythm and atrial fibrillation for transcriptomic analysis.

	Sinus Rhythm		Atrial Fibrillation	
	Male	Female	Male	Female
GSE41177	2	1	8	8
GSE79768	2	4	3	4
Total	4	5	11	12

**Table A2:** Clinical characteristics of patients for validation.

Characteristic	Sex		*p*
	Male, *n* = 35	Female, *n* = 39	
Age	65.00 ± 7.07	65.00 ± 8.45	ns
BMI	25.10 ± 3.36	25.14 ± 3.72	ns
Hypertension			ns
No	19 (54.29%)	19 (48.72%)	
Yes	16 (45.71%)	20 (51.28%)	
Diabetes			ns
No	32 (91.43%)	32 (82.05%)	
Yes	3 (8.57%)	7 (17.95%)	
Smoking			<0.001
No	10 (28.57%)	39 (100.00%)	
Yes	25 (71.43%)	0 (0.00%)	
Drinking			<0.001
No	12 (34.29%)	37 (94.87%)	
Yes	23 (65.71%)	2 (5.13%)	
LVEF (%)	61.96 ± 12.54	63.28 ± 7.43	ns
IVSd (cm)	1.00 ± 0.23	0.93 ± 0.14	ns
LVIDd (cm)	4.69 ± 0.55	4.56 ± 0.33	ns
LVPWd (cm)	0.95 ± 0.11	0.90 ± 0.12	ns
LVIDs (cm)	3.13 ± 0.59	3.00 ± 0.36	ns
AO-STJ (cm)	2.92 ± 0.30	2.71 ± 0.25	0.002
LA-ap (cm)	4.12 ± 0.51	4.24 ± 0.54	ns
eGFR, ml/(min·1.73 m^2^)	81.40 ± 15.02	79.16 ± 21.54	ns
TC, mmol/L	4.11 ± 0.90	4.50 ± 1.02	ns
TG, mmol/L	1.34 ± 0.59	1.46 ± 0.59	ns
HDL-C, mmol/L	1.23 ± 0.25	1.27 ± 0.26	ns
LDL-C, mmol/L	2.13 ± 0.66	2.38 ± 0.78	ns
AST, U/L	27.00 (18.00, 35.00)	24.00 (17.00, 37.00)	ns
ALT, U/L	24.00 (18.00, 32.00)	22.00 (17.00, 27.25)	ns

ns, not significant; BMI, body mass index; LVEF, left ventricular ejection fraction; IVSd, end-diastolic interventricular septum thickness; LVIDd, left ventricular end-diastolic inner diameter; LVPWd, left ventricular end-diastolic posterior wall thickness; LVIDs, left ventricular end-systolic inner diameter; AO-STJ, aorta sinotubular junction; LA-ap, anterior and posterior diameter of left atrium; eGFR, estimated glomerular filtration rate; TC, total cholesterol; TG, triglyceride; HDL-C, high-density lipoprotein cholesterol; LDL-C, low-density lipoprotein cholesterol; AST, aspartate aminotransferase; ALT, alanine aminotransferase.
